# P02-06 Citizen science during Covid-19 pandemic to enhance an activating environment in a low-SES neighborhood

**DOI:** 10.1093/eurpub/ckac095.025

**Published:** 2022-08-29

**Authors:** Berry Van Holland, Nikki Jepkema, Johan De Jong

**Affiliations:** School of Sports Studies, Hanze University of Applied Sciences, Groningen, The Netherlands

**Keywords:** citizen science, social innovation, community of practice, active lifestyle

## Abstract

It is widely known that people from a low-SES background show a less healthy and active lifestyle. One neighborhood in Groningen, the Netherlands, is a neighborhood housing about 12,000 citizens with on average a low-SES background. In the past, initiatives have been undertaken to promote an active lifestyle by implementing outdoor facilities stimulating physical activity. However, use of these facilities was poor due to lack of citizen involvement. Aim of this project was to engage citizens in the overall process of capturing, plan making and prototyping of concepts for an exercise-friendly physical and social environment.

In the period from January-November 2020 a Living Lab was set up following the “Our Voice” citizen science method. Participatory citizen science was applied in which a community of stakeholders (public and private parties) and citizens was set up. This composes the first step of design thinking: empathizing. The community addressed the aforementioned problem by creating more insight in promoting or degrading features in the neighborhood concerning an active lifestyle (design thinking step 2: defining). For this, citizens made use of the Stanford Neighborhood Discovery Tool. Due to local COVID-19 restrictions, citizens did not collect data individually but were accompanied by a researcher during research walks. The Tool allowed for systematic observations of the physical environment. Additionally, the emergent research walks gave additional information on neighborhood barriers and facilitators next to Discovery Tool data.

Use of the Discovery Tool created an overview of the neighborhood. Based on positive and negative features, new ideas were generated for improving exercise-friendliness (design thinking steps 3 and 4: ideating and prototyping). Furthermore, a work group of citizens was formed which discussed their prototypes with the local government and will be involved in carrying out the ideas.

Our project resulted in a citizen science approach which can be transferred to other neighborhoods. Use of the Discovery Tool showed many benefits for plan making for the neighborhood. Early and continuous involvement of citizens will lead to more sustainable engagement and is a powerful method to create engagement around societal problems and social innovation in the field of Health Enhancing Physical Activity.

